# Simple Syringe Method for Transfusion of Blood

**Published:** 1915-02

**Authors:** 


**Simple Syringe Method for Transfusion of Blood.** Abraham Zingher of N. Y., "Arch of Ped.," December, 1914, advises the use of a couple of 20-30 c.c. all-glass syringes. The arm of the donor is constricted with a rubber tubing clamped by an artery forceps, and the blood is withdrawn in turn from the median basilic and median cephalic veins of the two arms. The blood is injected immediately into the muscle, as the gluteals, not into subcutaneous tissue. Sterilization is secured by alcohol or iodine. Absorption of serum takes place rapidly, that of the corpuscular contents is supposed to occur within a day or two. 200-300 c.c. can be transfused at a sitting without inconvenience. (Note: For diagnostic withdrawal of blood, an ordinary rubber band serves the purpose.)

